# Is artificial intelligence magic dust for big-science facilities?

**DOI:** 10.1107/S2052252519016476

**Published:** 2020-01-01

**Authors:** Heloisa N. Bordallo, Christina Lioma, Jonathan Taylor, Dimitri N. Argyriou

**Affiliations:** a Niels Bohr Institute, University of Copenhagen, Universitetsparken 5, 2100 Copenhagen Ø, Denmark; b European Spallation Source ERIC, PO Box 176, SE-221 00 Lund, Sweden; c Department of Computer Science, University of Copenhagen, Universitetsparken 5, 2100 Copenhagen Ø, Denmark; d Data Managment and Software Center, European Spallation Source ERIC, PO Box 176, SE-221 00 Lund, Sweden

**Keywords:** artificial intelligence, big-science facilities, editorial

## Abstract

AI is no magic dust: for it to become a true discovery accelerator, much work is needed to make it transparent and robust.

*‘Siri^TM^, how do I make a room temperature superconductor?’ [Feel free to substitute your unsolved science grand challenge.]*

Many of us, including scientists, harbour the notion that artificial intelligence (AI) will gather the data we have today, sprinkle magic dust over them, and give us answers to scientific challenges that have kept us occupied for decades.

Of course, there is no magic dust. But perhaps due to its own success, science has reached a point where it could do with some. The sheer volume of scientific data in all their guises are now exceeding our capacity to extract knowledge and insight from those data in a timely fashion. This challenge is particularly acute at large scientific facilities, such as particle accelerators, and X-ray and neutron sources.

Today, AI can recognize a cat in a photo at least as well as we can. This astonishing capability has substantial implications for dealing with the data deluge. But ultimately, science is about gaining knowledge. We want to know *how* our brain recognizes a cat. This calls for a deeper understanding of AI as a tool for discovery and knowledge.

Against the backdrop of transformative developments in AI, it is of no surprise that the US Department of Energy, one of the world’s largest funders of science and big-science facilities, is planning to request $3–4 billion over 10 years to develop AI to accelerate scientific discovery (Service, 2019 ▸). While this investment would still be small compared with the private investments that have brought us technologies like Apple’s Siri and Amazon’s Alexa, it would be one of the first concerted efforts to develop AI to accelerate scientific discovery. And the bidding has not stopped; there is now talk of an additional $300 billion shot into the arm of AI by the United States government.

The impact of AI in harnessing the data deluge and accelerating discovery at neutron and photon sources was under intense discussion at a recent international workshop in Copenhagen.^1^ There was no doubt at the meeting that the enormous increase in the performance of neutron and X-ray sources, combined with advanced developments in optics and detectors, is providing science with unparalleled access to the inner workings of molecules and materials. However, these advances also expose a lack of investment in data storage hardware, as well as powerful software tools able to extract scientific knowledge and insight from data. The investment deficiency is so serious that it has become a bottleneck in using these facilities to their full potential.

Generating a data hoard is a mark of the success of big-science facilities, but can we really use AI to bring scientific understanding from the data these facilities generate? Through the discussions at the Niels Bohr Institute in Copenhagen, several AI related issues became clear. These issues challenge our perception of what are ‘good’ data, how much data we really need and finally how we extract scientific knowledge from data.

*Data, do we need them all?* Modern scattering instruments are rapidly reaching the milestone of collecting a PB of data per day, often in a single experimental run. This is driven as much by advanced detector technologies, as by the modern trend in scattering experiments to measure everything that is possible to measure in the energy-momentum space, *S*(*Q*,ω). Only a small fraction of the collected data, however, ultimately contributes to a publication or result. One approach is to minimize the data burden by distinguishing which data are actually measured or kept. Here AI can be a helpful and objective utility to make these choices. For example, by training an AI algorithm from previous experiments, or simulations, from the sample of interest, suggestions on the likely regions of interest to record the data with optimal data-collection parameters would help design and configure the experiment. In this context, objectivity is needed and this approach will require some detachment from previous data-collecting practices.

*What are ‘good’ data?* Experimentalists purposefully set up measurements where only one parameter is controlled, while others are held constant. To collect good data, we ensure that counting statistics for the measurements are sufficient for reliable inference, which usually means overcounting. This time-consuming approach requires good experimental stability, stable sample environments, reliability and ample beam time. If these conditions are not met, we label our data as bad data. However, recent examples at FELs have challenged what we call good data. Unsupervised AI algorithms have successfully extracted meaningful and insightful results from data whose measurement conditions may vary at random, as long as these conditions are recorded. Indeed, for some FEL applications, AI has been an irreplaceable tool for scientific success, extracting meaning from data with significant measurement uncertainties, due, for example, to beam jitter. This opens the question of what are in fact good data and how far can we use AI to construct an efficient and effective measuring protocol that can produce meaningful and robust scientific measurements.

*Automation and objectivity in data reduction and modelling.* A critical step in a scattering experiment is to reduce and correct the data into meaningful physical units amenable to analysis, and the determination of real physical or chemical variables. Often this step is routine, but it does hide choices that can fundamentally bias the reduced data. Another challenge is the sheer volume of the data, which can lead to ‘inelegant reduction’. Bulk reduction techniques are often black boxes that can make or break a successful experiment. A well trained AI can be a helpful accelerator, both in choosing the right parameters for data reduction, and for modelling within a data-rich environment.

*Interpretability.* In science, we seek understanding and insight. Any application of AI in a discovery environment would need to have interpretability and traceability to the choices it has made. For example, which part of the data were important for the AI to decide between two models, and does that make scientific sense? This is central to the potential impact of AI on scientific discovery. Further, interpretability and traceability must also highlight the converse: which part of the data are not relevant or not contributing to making a wider set of connections to models, as these may represent new gaps in knowledge that require further investigation. Ultimately, we need to *understand* (and control) the mechanisms, which underlie the behaviour of the system we are interested in.

Big-science facilities enable scientific discovery and hold the key to vast amounts of complex and diverse data. But, AI is no magic dust and for it to become a true discovery accelerator, much work is needed to make it transparent and robust. The investment needed by big-science facilities to make AI work for them is significant, especially in an environment where resources are squeezed and expertise hard to attract. There is no doubt that applying AI technology to hard won data will be invaluable for the broader society. But no single facility alone can take on this task, and collaboration with universities and others in the open-source community are essential in any strategy moving forward.

## Footnotes

1 ‘Workshop on Perspectives and Applications of Deep Learning for Accelerated Scientific Discovery at next generation X-ray and Neutron Sources’ organized by Heloisa N. Bordallo, Christina Lioma, Jon Taylor and Dimitri Argyriou (ESS) held on 26–27 September 2019 ▸ in Copenhagen, Denmark, https://indico.nbi.ku.dk/event/1256/.
